# Hidradenocarcinoma of the lower lip with squamoid differentiation: First reported case treated with Mohs micrographic surgery

**DOI:** 10.1016/j.jdcr.2026.08.053

**Published:** 2026-09-06

**Authors:** Henry Lim, Alyssa Forsyth, Marshall Hall, Steven Richardson, Dustin V. Wilkes, Stephen Weis, Stanislav N. Tolkachjov

**Affiliations:** a Department of Dermatology, HCA Medical City Fort Worth, Fort Worth, Texas; b University of North Texas Health Science Center, Texas College of Osteopathic Medicine, Fort Worth, Texas; c Division of Dermatology, Baylor University Medical Center, Dallas, Texas; d Texas A&M School of Medicine, Dallas, Texas; e Epiphany Dermatology, Dallas, Texas; f Department of Dermatology, University of Texas at Southwestern, Dallas, Texas

**Keywords:** adnexal, crateriform squamous cell carcinoma, hidradenocarcinoma, malignant eccrine acrospiroma, malignant hidradenoma, Mohs micrographic surgery, skin cancer, sweat gland carcinoma

## Introduction

Hidradenocarcinoma is a rare malignant adnexal ductal sweat gland tumor.^1^ It most commonly arises in middle-aged to older adults, with a median age of approximately 60 years.^1^ While most cases occur in the head and neck region, lip involvement is infrequently found.^2^ Mohs micrographic surgery (MMS) has attained favorable local control of hidradenocarcinoma, with a Mayo Clinic study of 10 patients reporting no recurrences after a mean follow-up of 7 years.^3^ We present a case of hidradenocarcinoma on the lower vermilion lip with dermoscopic findings. There has been a single report of hidradenoma of the lower lip and isolated reports of hidradenocarcinoma on the hair-bearing cutaneous or unspecified lip treated with excision. To our knowledge, this is the first reported case of hidradenocarcinoma affecting the vermilion lip treated with MMS.^2^

## Case presentation

A 41-year-old white, non-Hispanic male with a history of mild intellectual and developmental disability presented with a 2-month history of a rapidly growing lower lip lesion. The lesion was pruritic and bled upon contact, such as when brushing his teeth. He denied pain, discharge, odynophagia, dysphagia, or dysphonia. He had been treated with topical acyclovir by another clinician for suspected herpes labialis, which did not improve the lesion. There is no personal or family history of skin cancer, radiation therapy, or head, neck, or thyroid malignancy.

Physical examination revealed a 1.5 cm nodule on the lower cutaneous lip with polymorphic vessels and superficial ulcerations that bled on contact. Superior to the nodule, there was a 1.2 cm flat plaque (Fig 1). Dermoscopy of the nodule and plaque highlighted a pink-to-red background with white structureless areas. There were irregular linear and dotted vessels surrounding and traversing the lesion (Figs 2 and 3). The lesion was mobile and not fixed to underlying bone or muscle. Bimanual palpation of the floor of the mouth was unremarkable. No submental, submandibular, or cervical lymphadenopathy was appreciated bilaterally.

**Fig 1:**
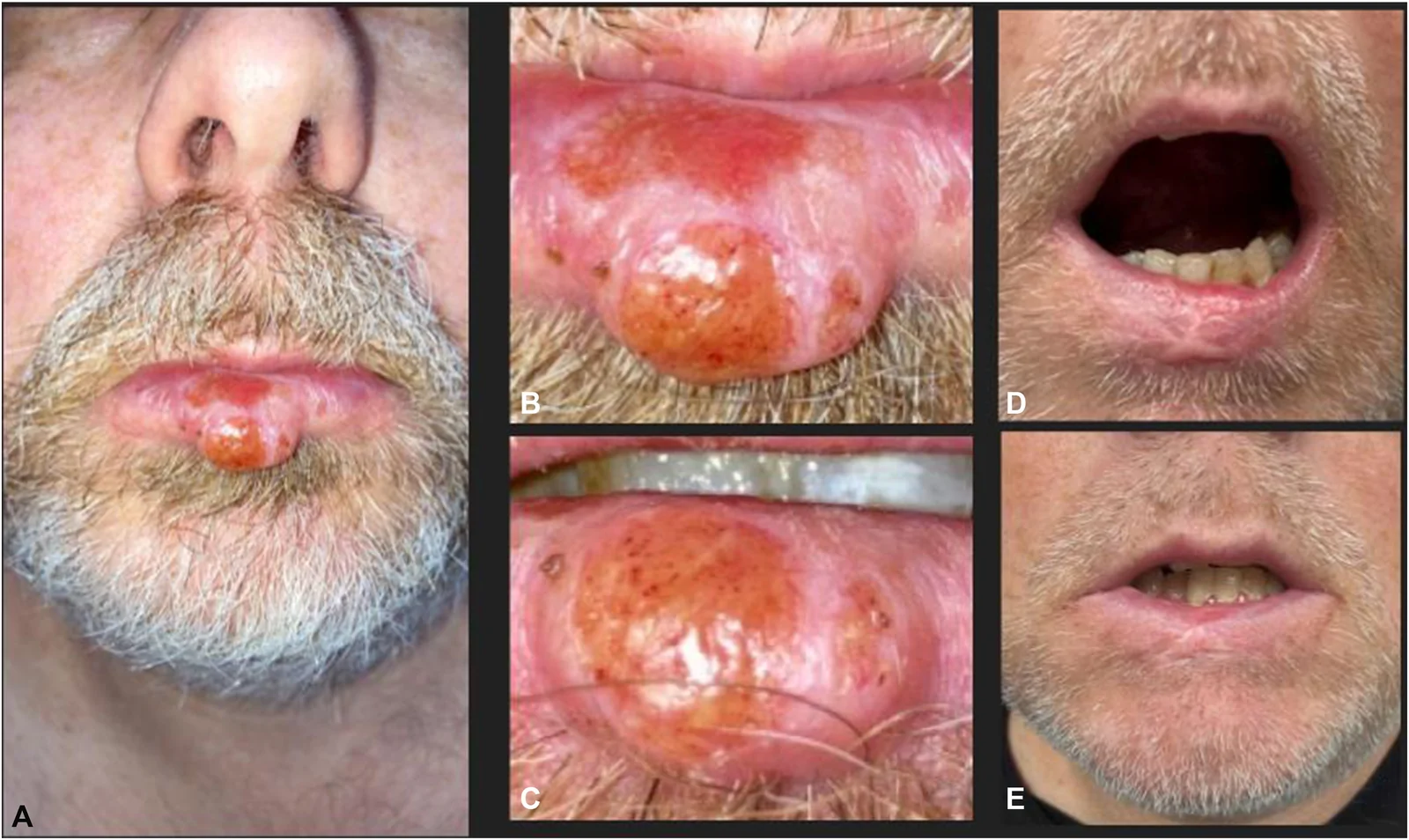
Clinical findings. **A,** 1.5 cm ulcerated nodule on the lower cutaneous lip, extending across the vermilion border. **B** and **C,** Superior to the nodule, there is a 1.2 cm pink-red plaque on the lower vermilion lip. **D,** 6 months post Mohs surgery and reconstruction with a split orbicularis myomucosal flap and reasonable functional outcome. **E,** 3 years recurrence-free. Note the lack of microstomia.

**Fig 2:**
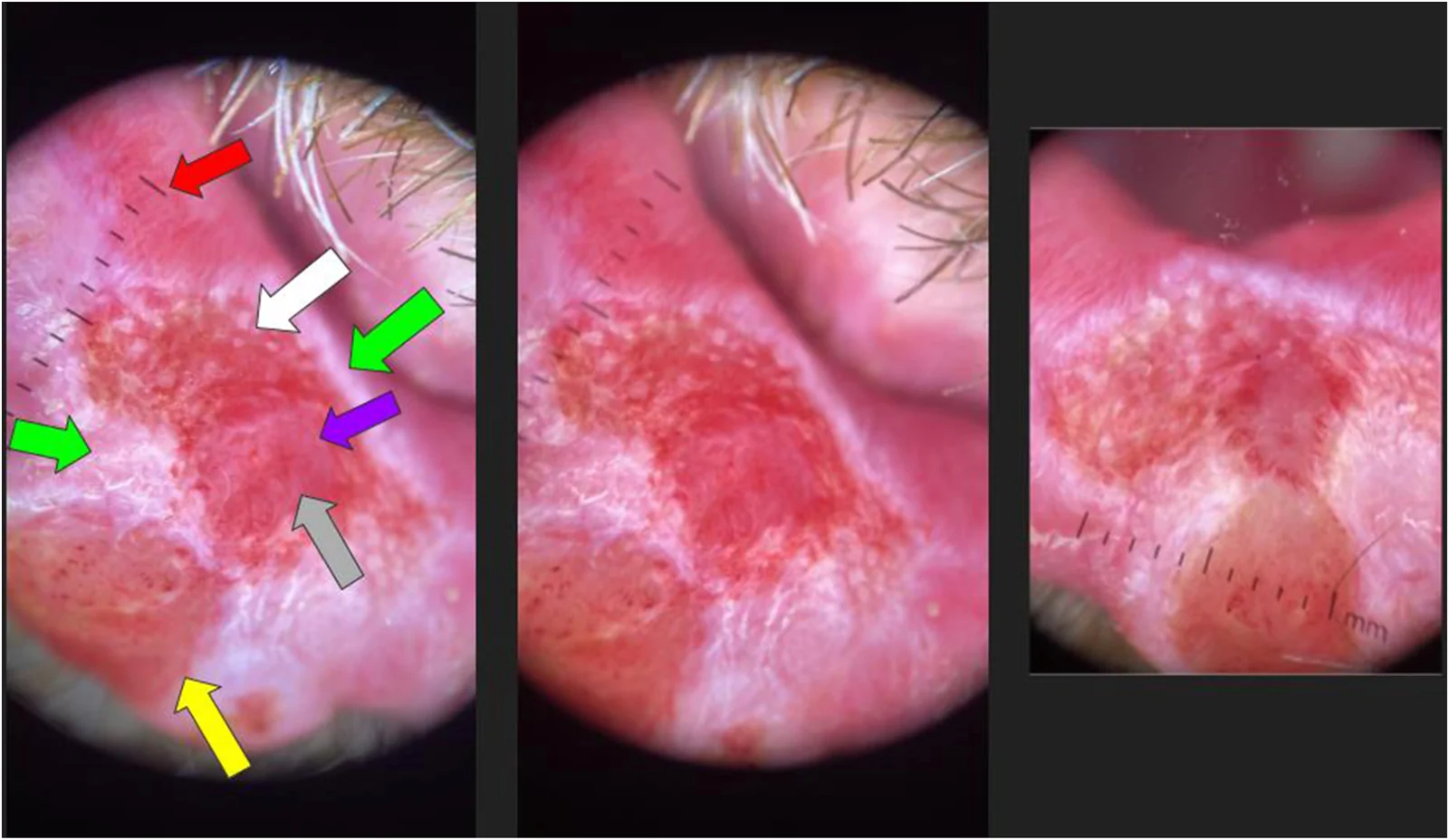
Dermoscopy of the lower lip plaque. There is a pink plaque (*gray arrow*) with a well-circumscribed orange-pink inferior nodule (*yellow arrow*), milky-red structureless area (*purple arrow*), and a dark pink superior red reticulated structureless area (red arrow). Pale pink-to-white bands circumscribe the ulcerations and plaque (*green arrow*). White crystalline structures can be appreciated throughout the lesion (*white arrow*).

**Fig 3:**
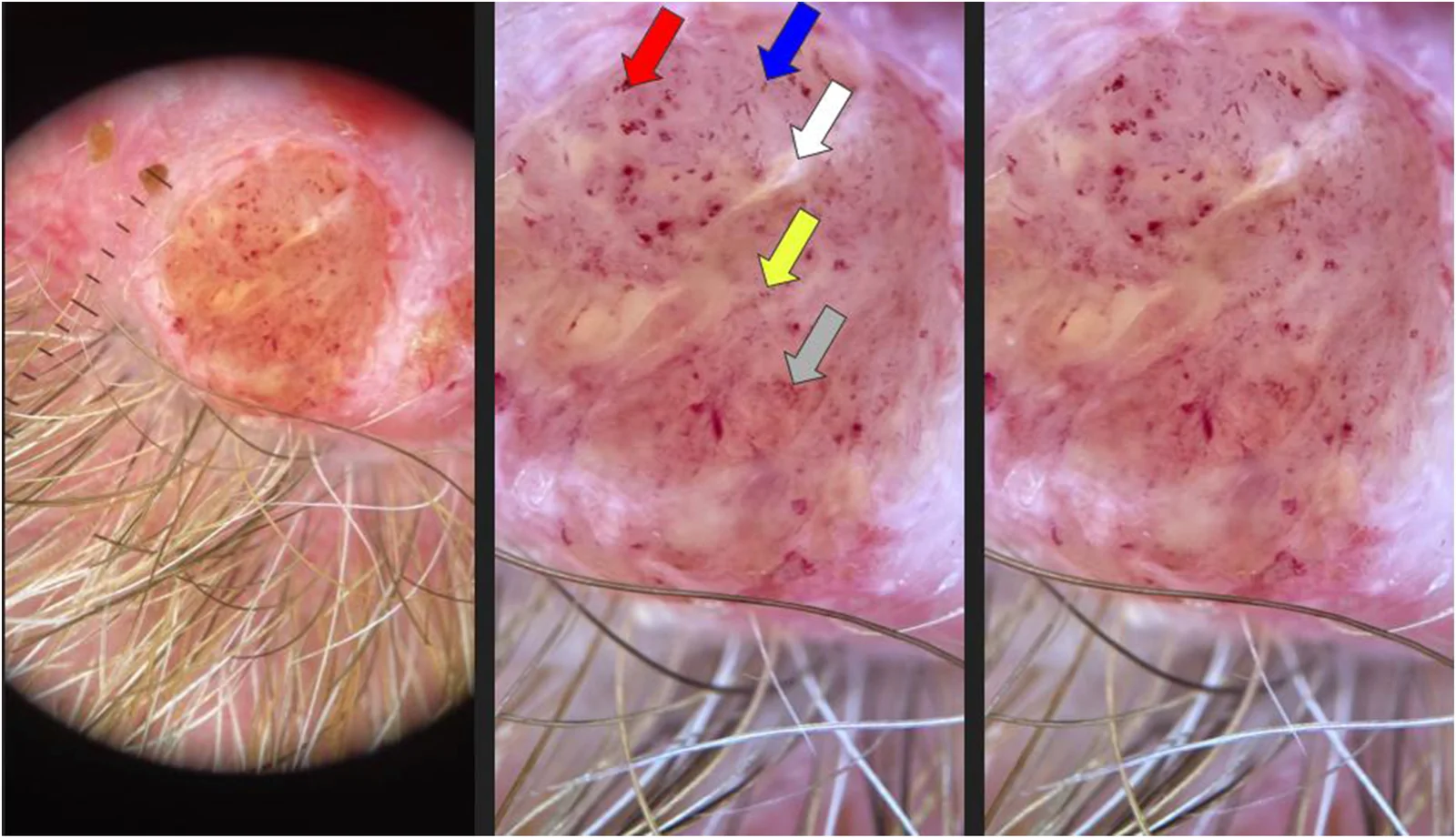
Dermoscopy the lower lip nodule. There is a well-circumscribed orange-yellow nodule. Polymorphous vessels are observed peripherally and across the involved area. There are irregular (*blue arrow*), glomerular (*red arrow*), dotted (*yellow arrow*), and corkscrew (*gray arrow*) vessels present. Shiny white streaks can also be appreciated (*white arrow*).

Histopathologic examination of a sample from the right inferior lower lip showed an epithelial neoplasm with adnexal differentiation, including areas consistent with clear cell hidradenoma and adjacent atypical zones showing scattered mitotic figures and pleomorphism, consistent with hidradenocarcinoma ex hidradenoma. A separate biopsy from the right superior lower lip revealed an irregular central keratin mass within a crateriform, acanthotic epidermis with foci of hyperchromatic, pleomorphic keratinocytes exhibiting incomplete maturation, parakeratosis, and individual cell keratinization, consistent with squamoid differentiation (Fig 4). In the clinical context, the pathology was consistent with a hidradenocarcinoma with squamoid differentiation.

**Fig 4:**
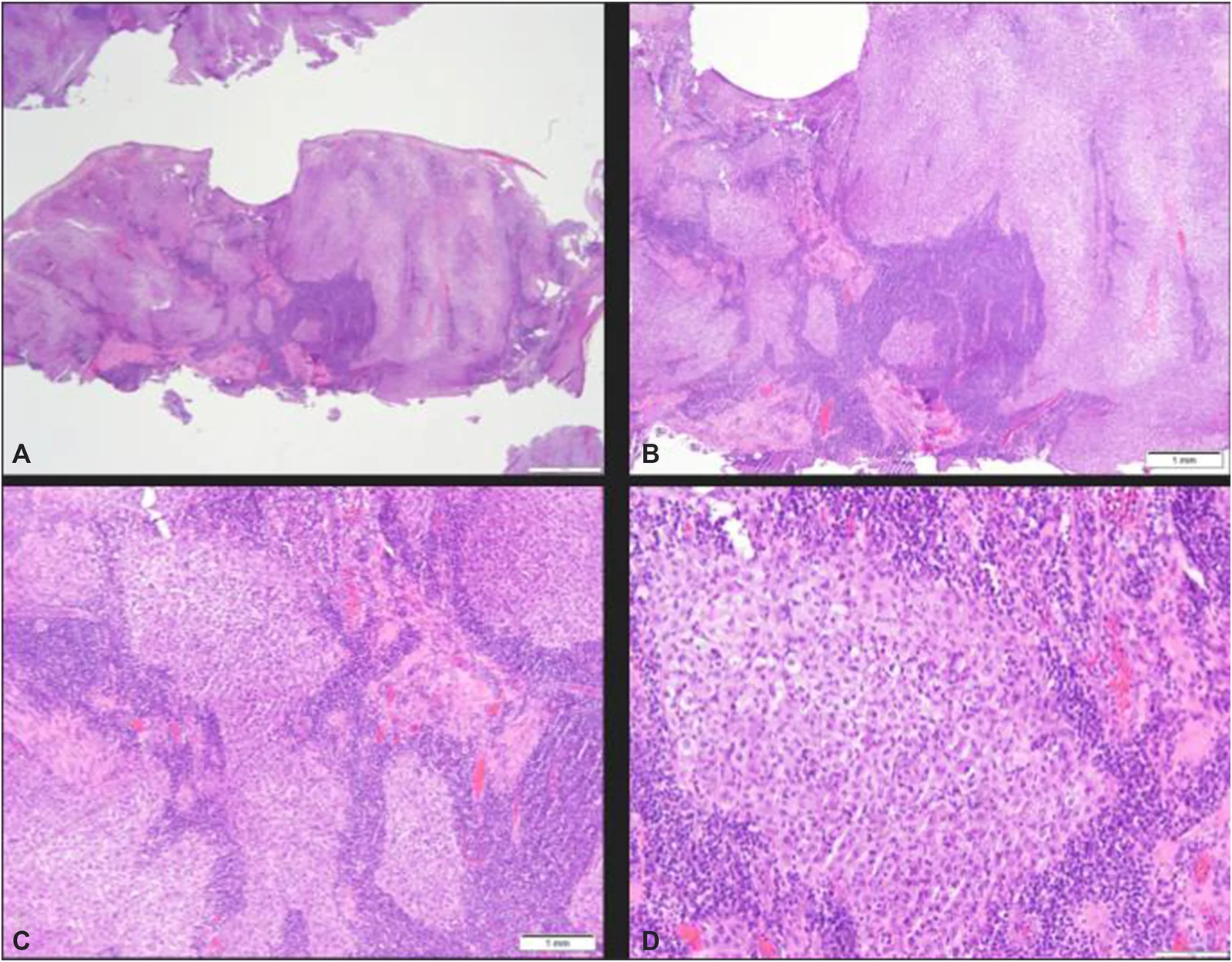
Biopsy of hematoxylin-eosin (H&E) stained Hidradenocarcinoma prepared via formalin-fixed-paraffin-embedding (FFPE). **A,** (2×) and **B,** (4×): A dermal infiltrating epithelial neoplasm with adnexal differentiation. **C,** (10×): Eccrine ducts within the nodules and prominent clear cells are present. **D,** (20×): Adjacent atypical zones showing scattered mitotic figures and pleomorphism.

Preoperative imaging showed no evidence of regional lymphadenopathy, local invasion, or distant metastatic disease. The tumor was successfully treated with MMS, requiring one stage. At 3-year follow-up visit, the patient remains free of recurrence.

## Discussion

Hidradenocarcinoma is an exceptionally rare malignancy with poorly defined survival.^1^ There is heterogeneity in reporting, as some studies describe favorable prognoses while others portray hidradenocarcinoma as an aggressive malignancy with poor morbidity and mortality.^4^^,^^5^ However, it is believed that long-term survival correlates with tumor grade.^1^ High-grade tumors can be aggressive with high rates of recurrence and metastasis, whereas low-grade tumors tend to yield a much more indolent course.^1^^,^^4^

Dermoscopy may guide evaluation, though distinctive diagnostic criteria are lacking. To date, only 3 other case reports have described dermoscopic findings in hidradenocarcinoma. Bakar & Ince reported a case of hidradenocarcinoma that exhibited polymorphic vessels, including dotted, irregular linear, hairpin, and glomerular types, on a pink and white background.^6^ Akay et al similarly described dotted vessels and white and pink structureless areas.^7^ Li et al found arborizing telangiectasias on dermoscopy of a hidradenocarcinoma on the right scapula.^8^ In this case, the dermoscopy of the lesion indeed exhibited pink areas with white structureless regions surrounding erosions and polymorphic vessels, including irregular linear and dotted types. However, these features are not specific to hidradenocarcinoma and may also be seen in tumors like amelanotic melanoma and squamous cell carcinoma.^6^^,^^7^

At the microscopic level, hidradenocarcinomas share overlapping morphological features with many other adnexal, cutaneous, and metastatic tumors, which can make diagnosis especially challenging. For instance, it may exhibit areas of squamoid differentiation, which could account for the concurrent findings of hidradenocarcinoma and squamous cell carcinoma-like features in this case.^9^ The overlap stems from the multipotent nature of sweat gland epithelium, which can differentiate along multiple lineages within a single tumor.^9^ This translates to a complex histopathological picture, wherein small or superficial biopsies may result in sampling error and an incorrect histologic diagnosis.^9^ This conundrum is illustrated in the present case, in which 2 separate biopsies of the same lesion demonstrated features of distinct tumor types.

Universal treatment standards for hidradenocarcinoma are not defined in the literature.^4^ However, wide local excision and MMS have shown success.^3^^,^^10^ The recurrence rate for wide local excision ranges between 10% to 50%.^3^^,^^4^ MMS outcomes have a reported recurrence rate of 0%.^3^^,^^4^ Of note, available studies on surgical outcomes are limited by their small sample size. For instance, the MMS study included 10 subjects.^3^ More data comparing treatment outcomes are necessary to determine ideal management strategies. Adjuvant radiotherapy and chemotherapy have also been used in cases where complete surgical resection alone was not sufficient.^4^^,^^10^ The patient in this case underwent MMS and had no clinical evidence of recurrence or metastasis at the 3-year follow-up visit, supporting the efficacy of this treatment method.

## Conclusion

Although rare, hidradenocarcinoma should be considered in the differential diagnosis of nodular and ulcerated lesions. Diagnosis can be challenging due to histopathologic overlap with benign adnexal tumors and other malignancies. Management of these tumors is not well defined in the literature. We present a case that was managed with MMS that, 3 years after surgery, has no clinical evidence of recurrence or spread. MMS should be considered for managing hidradenocarcinoma due to its low recurrence rate and ability to conserve tissue, which is especially important in the head and neck region. Additionally, this is one of the few cases to describe dermoscopic findings of hidradenocarcinoma. Future studies should further clarify dermoscopic features and delineate treatment guidelines for these rare, potentially aggressive tumors.

### Declaration of generative AI and AI-assisted technologies in the writing process

No AI was used in the drafting, analysis, or revision of this manuscript.

## Conflicts of interest

Tolkachjov is an investigator/speaker for Bioventus, Kerecis, Boehringer Ingelheim, Regeneron, Disc Medicine, Leo Pharma, StimLabs, and CASTLE. Lim, Forsyth, Hall, Richardson, Wilkes, and Weis have no relevant COI to declare.
